# Role of FNAC in the diagnosis of intraosseous jaw lesions

**DOI:** 10.4317/medoral.20274

**Published:** 2015-02-07

**Authors:** Surbhi Goyal, Sonal Sharma, Mrinalini Kotru, Neelima Gupta

**Affiliations:** 1 MD, DNB. MD. MD. Department of Pathology, University College of Medical Sciences & GTB Hospital, Dilshad Garden, Delhi, India; 2 MS. Department of Otorhinolaryngology, University College of Medical Sciences& GTB Hospital, Dilshad Garden, Delhi, India

## Abstract

**Background:**

FNAC of intraosseous jaw lesions has not been widely utilized for diagnosis due to rarity and diversity of these lesions, limited experience and lack of well established cytological features. Aim of the study was to determine the role of FNAC in the diagnosis of intraosseous jaw swellings.

**Material and Methods:**

42 patients underwent FNAC over a period of 7 years (2007-2013), of which 37 (88.1%) aspirates were diagnostic. Histopathology correlation was available in 33 cases and diagnostic accuracy of FNAC was calculated.

**Results:**

Lesions were categorized into inflammatory 3, cysts/hamartomas 15 and neoplasms 19. Mandibular and maxillary involvement was seen in 21 and 16 patients respectively. Of these, benign cysts and malignant lesions were commonest, accounting for 27% lesions (10 cases) each. One case of cystic ameloblastoma was misdiagnosed as odontogenic cyst on cytology. Overall, sensitivity and specificity of FNAC were 94.7% and 100% respectively with a diagnostic accuracy of 97.3%. Definitive categorization of giant cell lesions, fibro-osseous lesions, odontogenic tumors and cystic lesions was not feasible on FNAC.

**Conclusions:**

FNAC is a simple, safe and minimally invasive first line investigation which can render an accurate preoperative diagnosis of intraosseous jaw lesions, especially the malignant ones in the light of clinic-radiological correlation.

**Key words:** Jaw swellings, intraosseous, FNAC.

## Introduction

Conventionally, fine needle aspiration cytology (FNAC) is frequently being used for the diagnosis of salivary gland and neck swellings, and thyroid masses (1). The efficacy of FNAC for diagnosis and treatment planning of intra-osseous jaw pathology has not been well established (2). The diagnosis of intraosseous jaw lesions is often problematic because of their proximity to tooth apices and neurovascular bundles. Many patients are frequently followed up for long periods to look for sequential radiographic changes, foregoing open biopsy for definitive histological diagnosis. Thus, at times significant or serious diagnoses are often delayed. FNAC can offer the clinician a conservative alternative to the more invasive procedures such as open biopsy, if a proper correlation with clinico-radiological findings is done. Keeping in view these considerations, this study was conducted to determine the role and efficacy of FNAC in the diagnosis of intra-osseous jaw lesions.

## Material and Methods

This is a retrospective study including all the patients who presented to FNA clinic with jaw swellings over a period of seven years (2007-2013). The study was approved by the Institutional Ethics Committee and the need to obtain informed consent was waived.

- Subject population

A total of 42 patients who had intraosseous jaw bone involvement on orthopantomogram/ X-ray underwent FNAC on outpatient basis. Of these, 37 (88.1%) aspirates were diagnostic and included in the study. Non-osseous jaw lesions were excluded from the study. Case records were retrieved from the archives and each patient’s age, sex, and pertinent clinical history were recorded. Routine radiographic or computed tomography findings were noted to aid in the diagnosis.

- Fine needle aspiration (FNA)

Aspirates were taken with patient in supine or in sitting position with head support. No pre medication or local anesthesia was given to the patients and the procedure was explained before performing. Under strict aseptic conditions, FNA was performed without radiological guidance with a 22-23 gauge needle attached to a10 ml disposable plastic syringe. Needle was introduced into the area of radio lucency with cortical thinning and was gently manipulated into various directions within the swelling while maintaining negative pressure. Air dried smears made from the aspirated material were stained by May Grunwald Giemsa stain. Smears fixed in 95% alcohol were stained with Papanicolaou stain. Ziehl Neelsen stain was done to look for acid fast bacilli (AFB) in suspected cases of tuberculosis. Periodic Acid Schiff (PAS) and Gram’s stain were done wherever required. Immunocytochemistry was performed using standard technique wherever required.

- Histopathological examination (HPE) 

Histopathology correlation was available in 33 cases where excision/curettage/ incisonal biopsy was done and diagnostic accuracy of FNAC was calculated.

## Results

Of a total of 69,567 cases of FNACs performed over a period of five years, we encountered only 42 intraosseous jaw FNACs. Ages of the patients ranged from 2.5 to 76 years ( Table 1) with male: female ratio being 1.1:1. Youngest patient was a two and a half year old boy and eldest patient was a 76 year old male having multiple myeloma. Lesions were categorized into inflammatory 3, cysts/hamartomas 15 and neoplastic 19. The cytological diagnosis, age of patient and site of involvement along with number of respective cases are shown in  Table 1. Mandibular involvement was seen in 21 patients, while rest 16 patients presented with maxillary swelling. Of these, benign cysts and malignant lesions were commonest, accounting for 27% lesions (10 cases) each.

**Table 1:**
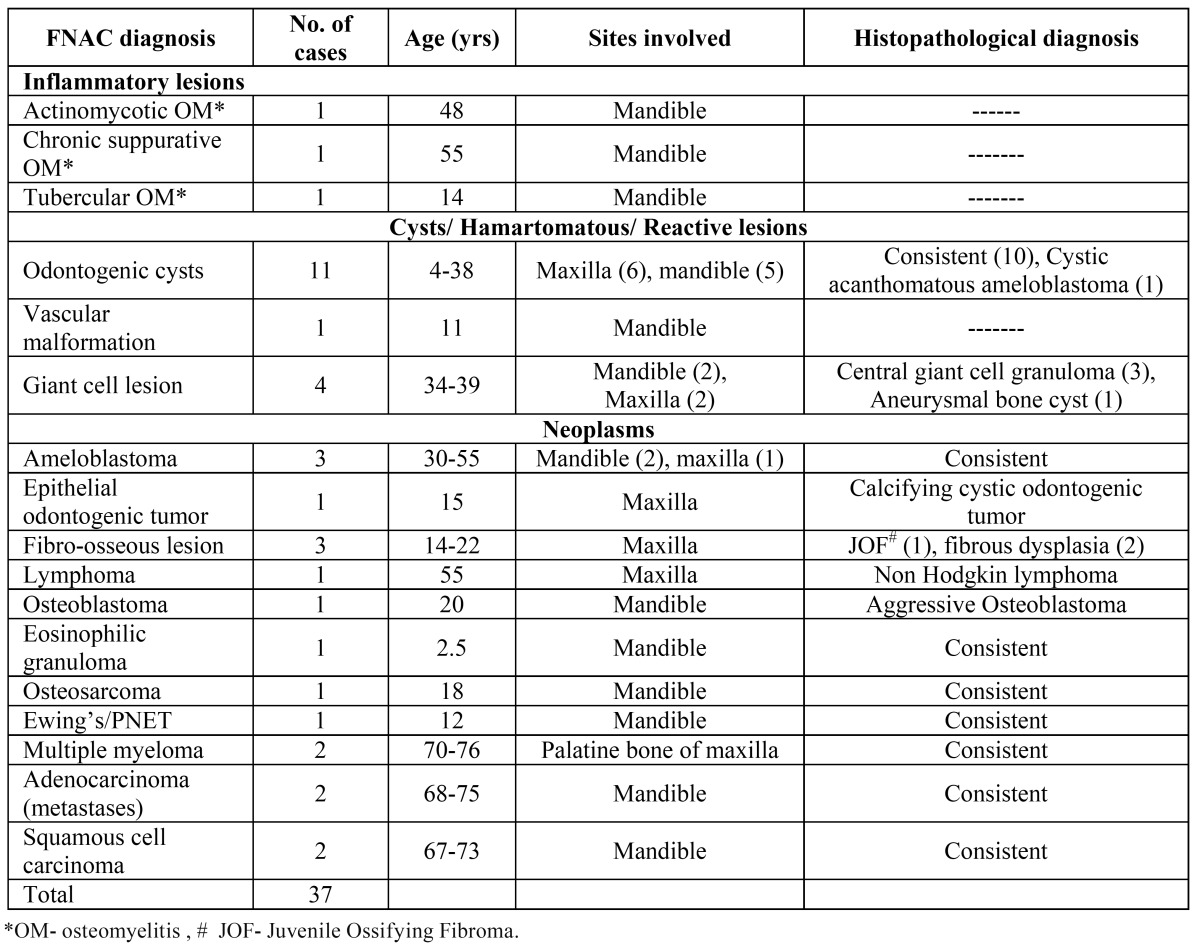
Clinicopathological features and cyto-histo correlation of jaw lesions.

**Figure 1:**
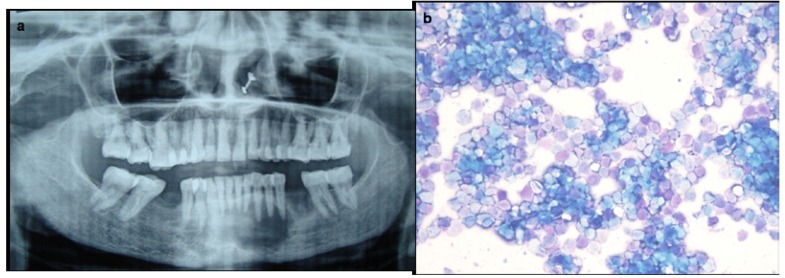
Orthopantomogram shows a well defined expansile lytic lesion in relation to roots of canines in mandible. b) Aspirate smear shows numerous anucleate squames suggestive of odontogenic cyst.

Aspirates from 11 patients yielded thick white necrotic fluid to clear straw colored fluid and showed numerous normal and anucleate squamous cells (Fig. 1). These cystic jaw lesions were reported as benign odontogenic cysts keeping in view the clinico-radiological findings. On histopathology, two were benign radicular cysts, four were dentigerous cyst and keratocyst each, and one turned out to be cystic acanthomatous ameloblastoma.

**Figure 2:**
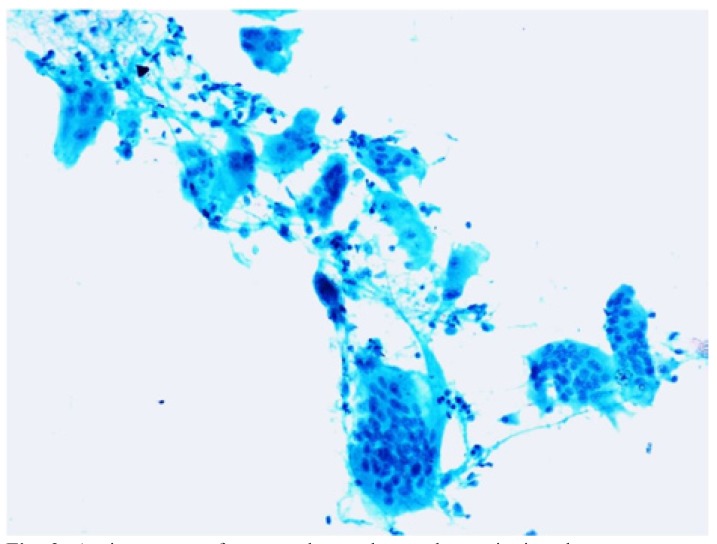
Aspirate smear from an ulcerated growth on gingiva shows numerous multinucleated giant cells and histiocytes adhered to fragments of fibrovascular stroma, suggestive of a giant cell lesion with the possibility of central giant cell granuloma (Pap x 400).

Aspirate smears from three middle aged females with jaw swelling revealed abundant met achromatic a cellular material along with fragments of spindle/stromal cells, inflammatory cells, histiocytes and scattered multi nucleated giant cells having 2-20 nuclei (Fig. 2). A diagnosis of giant cell lesion was given on FNAC, which on histopathology was confirmed as central giant cell granuloma (CGCG). FNAC yielded altered blood with numerous multi nucleated giant cells, hemosiderin laden macrophages and few stromal cells in a 20 year old boy, consistent with aneurysmal bone cyst (ABC) on radiology.

FNAC showed chronic inflammatory cells, necrotic debris and granulation tissue, features consistent with osteomyelitis (OM) on radiology. Epithelioid cell granulomas and AFB positivity in necrotic areas confirmed the diagnosis of tubercular OM in a 14 year child with past history of pulmonary tuberculosis. Blue filamentous structures surrounded by intense neutrophilic reaction on cytosmears were helpful in diagnosing actinomycotic OM.

**Figure 3:**
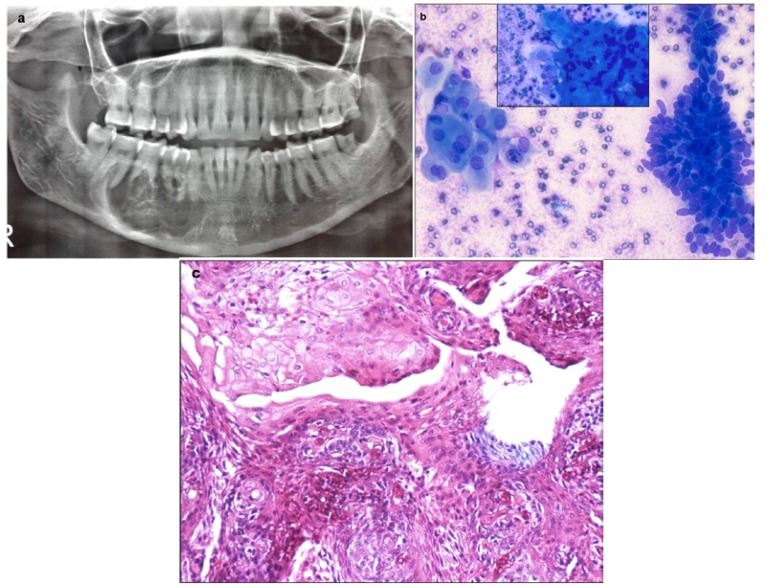
a) Orthopantomogram shows a large well-defined expansile lytic lesion involving the body of mandible, in bilateral paramidline regions. No cortical breech / periosteal reaction / tooth displacement / root resorption seen. b) Aspirate shows cohesive cluster of basaloid epithelial cells with peripheral palisading and polygonal squamous cells with dense inky blue cytoplasm (Inset) in a proteinaceous background, suggestive of ameloblastoma (MGG x 100). c) Histopathology confirms the squamous differentiation within the basaloid clusters (H&E x200).

**Figure 4:**
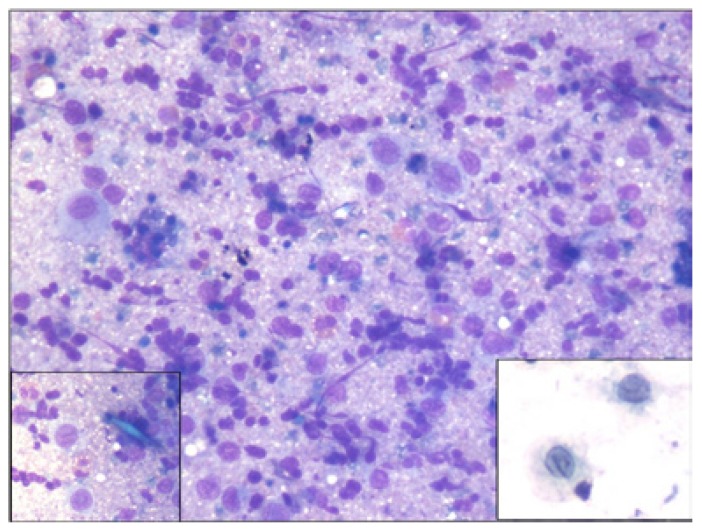
Aspirate from a case of eosinophilic granuloma involving mandible shows numerous histiocytes, eosinophils and neutrophils (MGG x400). Inset shows characteristic rhomboid blue Charcot Leyden crystals and Langerhans cell histiocytes with longitudinal nuclear grooves.

**Figure 5:**
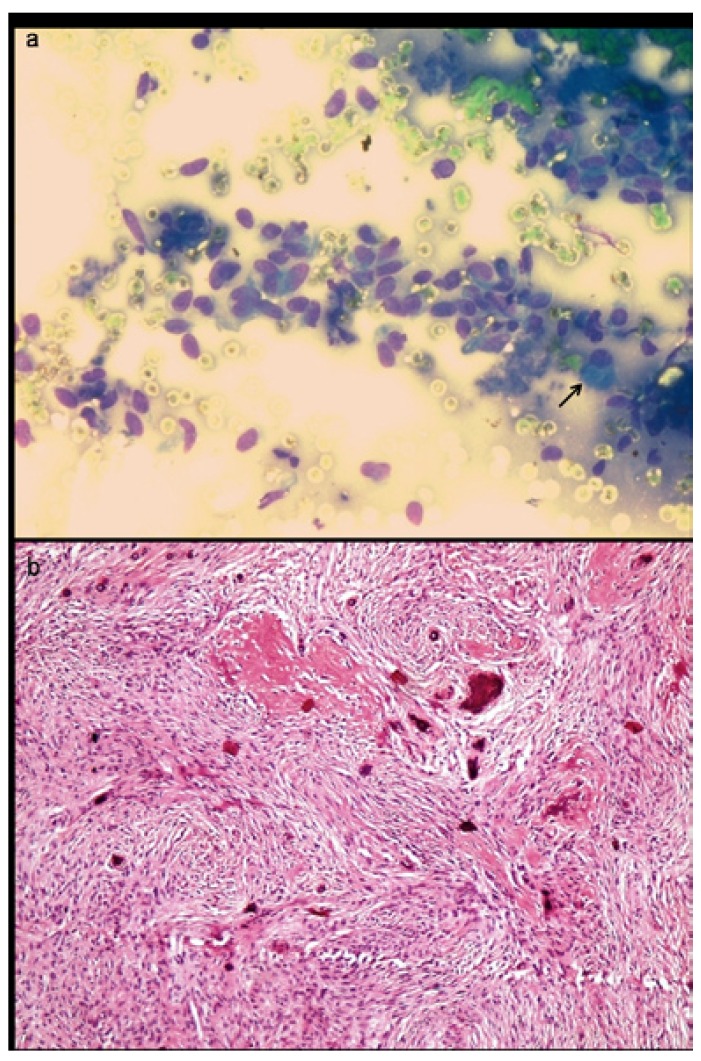
a) Aspirate smear from a mixed radiolucent lesion in maxilla shows presence of numerous plump fibroblastic cells and occasional osteoblast, suggestive of fibrosseous lesion (MGG x400). b) Histopathology shows predominantly fibroblastic stroma and irregular deposits of pink hyaline cementum like material with central calcification, confirming the diagnosis of cemento-ossifying fibroma (Hematoxylin & Eosin x100).

**Figure 6:**
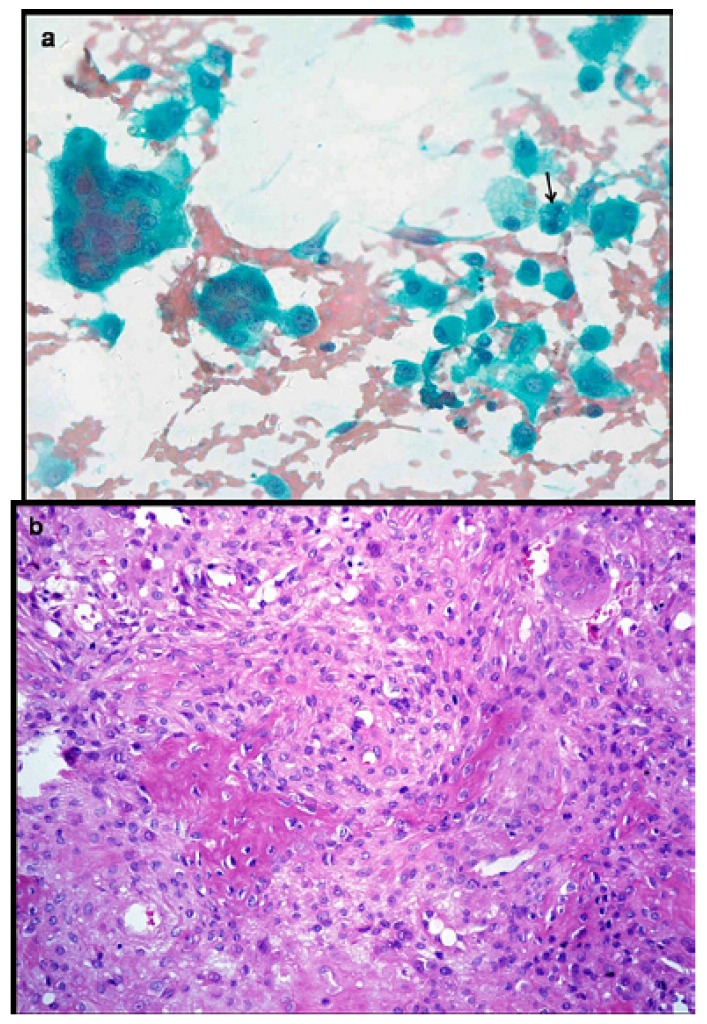
a) Aspirate smear shows plasmacytoid osteoblasts having moderate amount of cytoplasm with round nuclei, fine chromatin and distinct, single nucleolus. Few benign appearing osteoclastic giant cells and occasional binucleated cells are also seen (Pap x400). b) Histopathology confirms a bone forming tumor comprising osteoid surrounded by rim of epithelioid osteoblasts. Intervening loose fibrovascular stroma has scattered osteoclasts (H&E x200).

Three cases were reported as ameloblastoma (Fig. 3) and one case as epithelial odontogenic tumor with marked calcification on FNAC, which were confirmed on excision. FNAC from a lytic lesion in the ramus of mandible in a 2 year old boy yielded pus like material and smears showed numerous histiocytes with nuclear grooving, eosinophils and Charcot Leyden crystals, suggestive of eosinophilic granuloma (Fig. 4). Presence of numerous plump fibroblastic cells, osteoblasts and acellular calcified cementum like material were suggestive of fibro-osseous lesion on cytology (Fig. 5). Histopathology confirmed the diagnosis of cemento-ossifying fibroma, psammamatoid variant in a 14 year old girl and fibrous dysplasia in two other cases. Cytosmears showed plasmacytoid cells with moderate amount of cytoplasm along with fair number of benign appearing osteoclastic giant cells and scant amount of osteoid matrix in a 20 year old boy (Fig. 6). Radiological impression was of a primary bone tumor. FNAC diagnosis of an osteoblastoma was suggested which was confirmed on histopathology.

**Figure 7:**
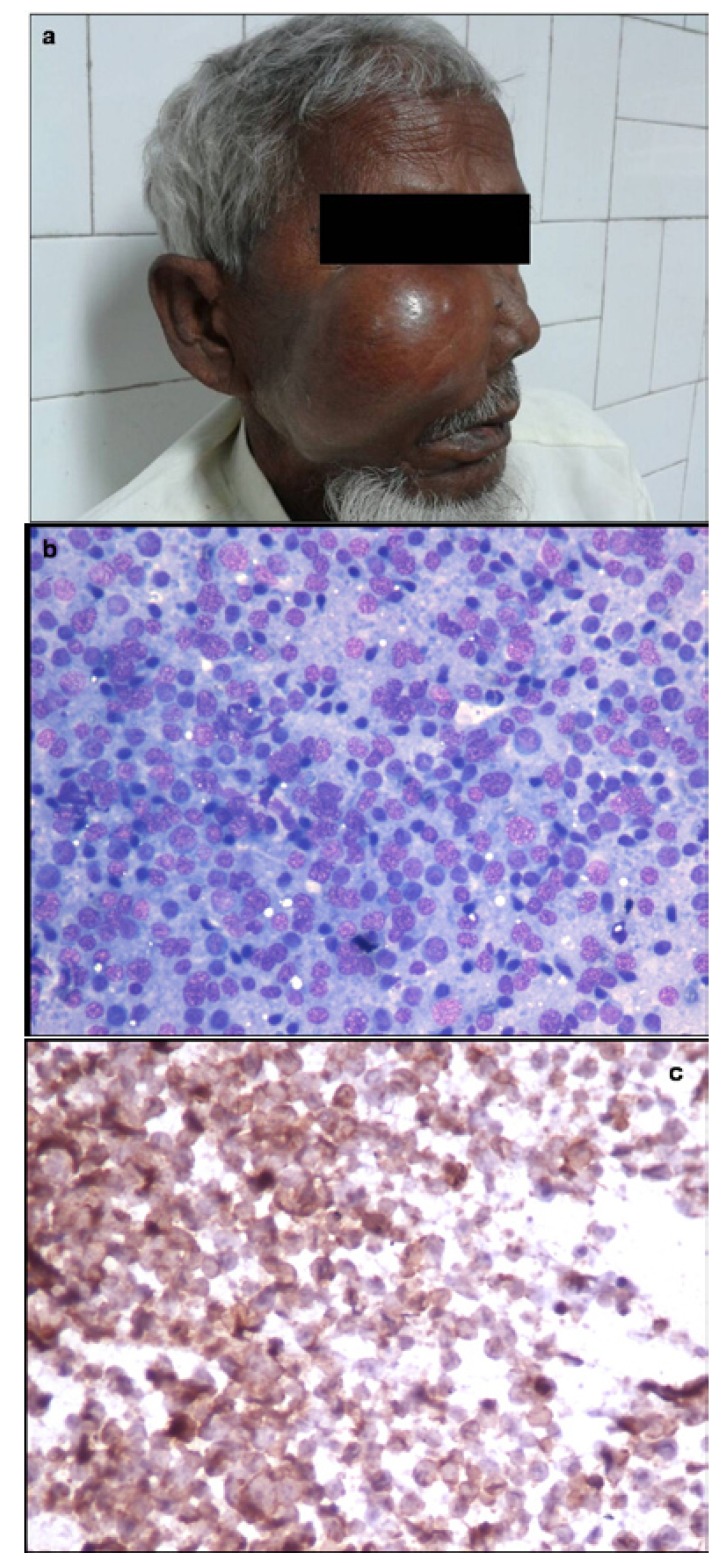
Photograph of a 55 year old patient presenting with right cheek mass. b) Aspirate smear shows features of Non Hodgkin lymphoma (MGGx 200) c) On immunocytochemistry, tumor cells were strongly CD20 positive (Immunostain x 200).

FNAC from a rapidly growing maxillary mass in a 55 year old male showed features suggestive of non Hodgkin lymphoma (NHL) and excluded the clinico-radiological diagnosis of metastases. On immunocytochemistry, the tumor cells were positive for CD20 and negative for CD3 (Fig. 7). Cytosmears from an 11 year old boy showed small round tumor cells with vacuolated cytoplasm which was PAS positive. Membranous positivity with CD99 on immunocytochemistry confirmed the diagnosis of Ewing’s/PNET. Aspirate from the mandibular swelling in two elderly patients showed primary squamous cell carcinoma with metastases into cervical lymph nodes. Two patients presented with multiple lytic lesions in mandible which were reported as metastases on FNAC. Of these, a 57 year old female was a diagnosed case of breast carcinoma and the other male patient on further work up was found to have lung carcinoma. Presence of malignant spindle cells with nuclear atypia in a background of met achromatic osteoid in a rapidly growing mass in an 18 year old boy clinched the diagnosis of osteosarcoma. Presence of sheets of mature and immature plasma cells in two elderly patients with history of bone pains and systemic complaints was diagnostic of multiple myeloma and excluded the possibility of metastases.

Diagnostic accuracy of FNAC

None of the intra-osseous jaw lesion was false positively diagnosed on FNAC and hence the specificity was found to be 100%. We had only one case of cystic ameloblastoma which was falsely diagnosed as odontogenic cyst. Therefore, specificity of FNAC was 94.7%. FNAC was 100% accurate in all the ten malignant lesions, which were confirmed on histopathology. Overall diagnostic accuracy of FNAC in our study was 97.3%.

## Discussion

Incisional biopsy has been considered as a prime diagnostic modality for preoperative presumptive diagnosis of intraosseous jaw lesions due to limited experience with FNAC, difficulty in accessing and peculiar anatomy of the maxillofacial region, diversity and rarity of these lesions and lack of well established cytological features. Very few studies have explored the role of FNAC for the diagnosis of jaw lesions (1-5). Extreme care and recognition of the limitations of cytology are essential in the evaluation of jaw FNAC.

Ramzy *et al*. (3) have studied FNAC of radio lucent lesions of jaws in 23 cases and concluded that FNAC is a valuable tool in differentiating benign and malignant lesions of the jaws. Thinning or destruction of cortical bone permits the use of thin needles for aspiration (4). Radio graphically a unilocular/multilocular radio lucent expansile lesion can be seen in odontogenic cyst, central giant cell granuloma, myxoma, ABC or cystic ameloblastoma (5).

Radiologically, osteomyelitis is seen as an ill defined osteolytic lesion with periosteal reaction and sclerosis and mimics Ewing’s tumor or a dento-alveolar abscess with bone destruction (6). Most cases of chronic suppurative osteomyelitis of jaw are caused by bacterial infection spreading from a contagious focus (6). Infection with Actinomyces is mostly of endogenous origin, however, there was no history of discharging sinus, trauma or surgery in our case, which misled the clinical diagnosis. The involvement of the mandible by tubercular infection is extremely rare as it contains less cancellous bone (7). Considering the high endemicity of tuberculosis in our population, the presence of AFB positivity, necrosis and epithelioid cell granulomas are indicative of tuberculosis unless proven otherwise (8). The same aspirate can be sent for micro biological culture sensitivity, to plan appropriate treatment. Therefore, utility of FNAC is in the early diagnosis and management of OM by excluding the differential diagnoses of neoplasms and invasive surgical intervention to obtain biopsy can be avoided.

The exact location, history and radiological appearance of cystic lesions of jaw must be considered before a definitive cytological diagnosis is made. Ramzy *et al*. reported that cystic lesions of the jaw bones lined by stratified squamous lining may pose a diagnostic problem (3). We encountered a case of cystic ameloblastoma, which was misdiagnosed as odontogenic cyst on cytology. On reviewing the slides, we found few cohesive clusters of epithelial cells with occasional peripheral palisading. The utility of FNAC in the diagnosis of ameloblastoma has been described by previous authors (9-12). The characteristic cohesive epithelial clusters of smaller basaloid cells with peripherally placed tall columnar cells and occasional large squamous cells aid in excluding the differential diagnosis of ameloblastoma (10,11).

FNAC plays a valuable role in the preoperative diagnosis and management of extremely vascular lesions like hemangiomas. Variable unilocular / multilocular radio lucent appearance on radiology often misleads the preoperative diagnosis (13,14). Free flowing fresh blood on aspiration should alert the pathologist and preclude biopsy/intervention in view of risk of severe hemorrhage.

Fibro-osseous lesions of the jaw represent a broad range of entities. We offered the cytological diagnosis of “benign fibro-osseous lesion” in our cases and the individual lesion was confirmed by histopathology only. Obtaining cellular aspirates may be quite difficult in heavily calcified lesions, as was seen in a case of cement-ossifying fibroma. Since these lesions are very firm and difficult to aspirate, inadequate aspirates often pose a major limitation for diagnosis in fibro-osseous lesions (2,15).

Giant cell lesions of the jaw include central giant cell granuloma (CGCG), giant cell tumour (GCT), aneurysmal bone cyst (ABC), traumatic bone cyst, cherubism, and brown tumour of hyperparathyroidism. Presence of giant cells and histiocytes adherent to stromal fragments in a young female with normal serum calcium, parathyroid hormone and phosphorus levels is strongly suggestive of CGCG (3). ABC is a non-neoplastic and non-odontogenic cyst, accounting for <2% cases in jaw bones (16). Radiological appearance of an eccentric, unilocular, radio lucent lesion with cortical expansion though suggestive of ABC, is not pathognomonic, and needs to be confirmed on histopathology (16). Role of cytology in the diagnosis of giant cell lesions of jaw has been mentioned by few authors (4,17).

FNAC is a very useful first line investigation to diagnose and type malignant lytic jaw bone lesions. If numerous eosinophils with Charcot-Leyden crystals and convoluted histiocytes with longitudinal grooves are identified in a child having solitary radiolucent intraosseous lesion, diagnosis of eosinophilic granuloma should be seriously considered. Likewise, diagnosis of lymphoma, plasmacytoma/multiple myeloma, Ewing´s/PNET, osteosarcoma, metastases and squamous cell carcinoma is usually straightforward on cytomears if cellular aspirates are available in a background of appropriate clinical setting. Though osteosarcoma is the most common malignant tumor of the jaw (18), we encountered only one case. Highly pleomorphic spindle and round cells with nuclear atypia along with osteoid and multinucleate giant cells help to differentiate from benign osteoblastoma (19). Metastasis to the jaw bones is very rare (accounts for only one percent of all oral malignancies), owing to the scant red marrow in mature jaw bone (20). Clinico-radiologically, it has nonspecific features and masquerades other odontogenic lesions leading to delayed diagnosis (21).

Use of supplementary ancillary techniques like immunocytochemistry can help in confirmation and further subtyping of tumors. Non Hodgkin Lymphoma (NHL) can be further categorized into B/T cell type as was possible in our case of diffuse large B cell lymphoma involving the maxilla. Membranous immunopositivity for CD99 narrowed the diagnosis to Ewing’s/ PNET within small round blue cell tumor category in a 11 year old child. FNAC supplemented with immunocytochemistry can serve as an alternative to biopsy in malignant intraosseous jaw lesions.

 In our study, the sensitivity, specificity and diagnostic accuracy of FNAC in jaw lesions were 94.7%, 100% and 97.3%, similar to that of previous studies (22,23). 12% aspirates were non-diagnostic in our study, mostly due to poor cellularity. We had only one false negative case of cystic acanthomatous ameloblastoma in our study, which was due to interpretation error, though the cellularity was adequate. Possibility of false negative diagnosis cannot be ruled out especially in case of uni/multicystic ameloblastoma due to inadequate aspirates (23). False negative/ inadequate FNAs may be due to cystic component, hemorrhage, excessive fibrosis, calcification and superimposed infection (1). Repeat aspiration from multiple sites in suspected cases of malignancy can be helpful. We did not encounter any major procedure related complications in the present study.

We found FNAC to be less sensitive for definitive diagnosis of benign pathological conditions of the jaw than for the malignant lesions, similar to that reported by Khan *et al*. (2). Lack of architectural context of the FNAC material, non representative sampling and nonspecific morphological features on cytosmears were the main limitations in definitive diagnosis, which we encountered in discrepant cases. For example, to distinguish a dentigerous cyst from an odontogenic keratocyst, it is not feasible to evaluate the characteristics of the lining cells on FNAC.

To conclude, the present study highlights the role of FNAC in the diagnosis of intraosseous jaw lesions. FNA is a simple, quick and cost effective procedure, which can be easily performed preoperatively in an outpatient department. It can obviate the need of open biopsy which is much more painful, requires local anesthesia and is associated with risk of bleeding. FNA was helpful in differentiating between inflammatory and neoplastic lesions, especially in diagnosis of malignant lesions. Overall diagnostic accuracy of FNAC was 97.3% in our study, with 100% specificity. In inflammatory lesions of infective etiology, FNAC was diagnostic and can aid in early implementation of therapy. FNAC can broadly diagnose giant cell lesions, fibro-osseous lesions, odontogenic tumors and cystic lesions; however, definitive categorization may not be always possible due to lack of specific cytomorphological features. Availability of quick staining methods and immunocytochemistry in a background of complete clinicoradiological correlation can provide a rapid and accurate preoperative diagnosis in most of the cases, guiding further management.
